# Effect of dried fruit on postprandial glycemia: a randomized acute-feeding trial

**DOI:** 10.1038/s41387-018-0066-5

**Published:** 2018-12-11

**Authors:** Effie Viguiliouk, Alexandra L Jenkins, Sonia Blanco Mejia, John L Sievenpiper, Cyril W C Kendall

**Affiliations:** 1 Toronto 3D Knowledge Synthesis and Clinical Trials Unit, St. Michael’s Hospital, Toronto, Canada; 2 Department of Nutritional Sciences, Faculty of Medicine, University of Toronto, Toronto, Canada; 3 Glycemic Index Laboratories, Toronto, Canada; 4 Division of Endocrinology & Metabolism, St. Michael’s Hospital, Toronto, Canada; 5 Li Ka Shing Knowledge Institute, St. Michael’s Hospital, Toronto, Canada; 6 College of Pharmacy and Nutrition, University of Saskatchewan, Saskatoon, Canada

## Abstract

**Background/Objectives:**

To investigate the effect of dried fruit in modifying postprandial glycemia, we assessed the ability of 4 dried fruits (dates, apricots, raisins, sultanas) to decrease postprandial glycemia through three mechanisms: a glycemic index (GI) effect, displacement effect, or ‘catalytic’ fructose effect.

**Subjects/Methods:**

We conducted an acute randomized, multiple-crossover trial in an outpatient setting in 10 healthy adults. Participants received 3 white bread control meals and 12 dried fruit test meals in random order. The test meals included each of 4 dried fruits (dates, apricots, raisins, sultanas) alone (GI effect), 4 of the dried fruits displacing half the available carbohydrate in white bread (displacement effect), or 4 of the dried fruits providing a small ‘catalytic’ dose (7.5 g) of fructose added to white bread (‘catalytic’ fructose effect). The protocol followed the ISO method for the determination of GI (ISO 26642:2010). The primary outcome was mean ± SEM GI (glucose scale) for ease of comparison across the three mechanisms.

**Results:**

Ten healthy participants (7 men, 3 women; mean ± SD age and BMI: 39 ± 12 years and 25 ± 2 kg/m^2^) were recruited and completed the trial. All dried fruit had a GI below that of white bread (GI = 71); however, only dried apricots (GI = 42 ± 5), raisins (GI = 55 ± 5), and sultanas (51 ± 4) showed a significant GI effect (*P* < 0.05). When displacing half the available carbohydrate in white bread, all dried fruit lowered the GI; however, only dried apricots (GI = 57 ± 5) showed a significant displacement effect (*P* = 0.025). None of the dried fruits showed a beneficial ‘catalytic’ fructose effect.

**Conclusions:**

In conclusion, dried fruits have a lower GI and reduce the glycemic response of white bread through displacement of half of the available carbohydrate. Longer-term randomized trials are needed to confirm whether dried fruit can contribute to sustainable improvements in glycemic control.

**Trial registration:**

ClinicalTrials.gov identifier, NCT02960373

## Introduction

Dried fruits show promising potential for blood glucose management. Previous trials conducted in individuals with and without diabetes have shown dried fruits (including dates, apricots, raisins, and sultanas) to have a low (≤55) to medium (56–69) glycemic index (GI)^1–7^ and to have beneficial effects on postprandial glucose^3,6,8–11^ and insulin^3,6,11^ levels, as well as HbA_1c_^8^ compared to high GI foods (e.g., crackers, cookies, white bread, glucose solution). However, the effect of combining dried fruits with high GI foods has not been adequately addressed. Current research suggests that combining dried fruits with high GI foods by displacing available carbohydrate may benefit postprandial glycemia in comparison to high GI foods alone. This is supported by acute studies showing that nuts and/or dried fruits combined with high GI foods can attenuate relative glycemic responses in healthy participants when compared to high GI foods alone^7,12,13^. Dried fruits may also benefit postprandial glycemia by providing small or ‘catalytic’ doses of fructose (≤10 g/meal). Fructose, through its metabolite fructose-1-P, has shown ‘catalytic’ effects on hepatic glucose metabolism through the induction of glucokinase activity in hepatocytes^14–17^. Infusion studies in humans have shown that this mechanism relates to a ~3-fold increase in glycogen synthesis under euglycemic conditions in healthy individuals^18^ and a ~30% decrease in hepatic glucose output under hyperglycemic conditions in individuals with type 2 diabetes^19^. Clinical translation of these findings have shown ‘catalytic’ doses of fructose at 7.5 and 10 g to decrease postprandial glycemic responses to oral glucose or high GI meals (e.g., mashed potatoes) by ~15–30% in healthy individuals^20,21^ and those with diabetes^22^.

Therefore, to address this knowledge gap, we aimed to: (1) quantify the GI of 4 different types of dried fruit (dates, apricots, raisins, sultanas) (GI effect); (2) to assess the ability of these 4 dried fruits to decrease the postprandial glycemic response to white bread by partially displacing available carbohydrate (displacement effect); and (3) by providing a ‘catalytic’ dose of fructose (‘catalytic’ fructose effect).

## Subjects and methods

### Participants

Participants were recruited from the Glycemic Index Laboratories clinic volunteer roster. Inclusion criteria consisted of men or non-pregnant women aged 18–75 years who were in good health. Individuals with a known history of diabetes, heart disease, liver disease, kidney disease, thyroid disease, HIV, or any other major illnesses that may affect carbohydrate metabolism, or using medications which might either make participation dangerous to the individual or affect the results were excluded.

The study was conducted in accordance with the Declaration of Helsinki and was approved by the Western Institutional Review Board® (protocol number: 971199) which meets all the requirements of the U.S. Food and Drug Administration (FDA), the Department of Health and Human Services (DHHS), the Canadian Health Protection Branch (HPB), Canadian Institutes of Health Research (CIHR) and the European Community Guidelines. All participants provided written informed consent prior to starting the study and received a financial reward for their participation. The study was registered on ClinicalTrials.gov (identifier: NCT02960373).

### Study design

This was an acute randomized, multiple-crossover trial that followed the International Organization for Standardization (ISO) method for determining GI (ISO 26642:2010). Eligible participants underwent 15 separate study meals on 15 separate occasions, with each participant undergoing up to 3 study meals per week separated by at least one day. Randomization of the sequence of study meals was performed using a computer random number generator by a coordinator blinded to the treatment allocation. On each test day, participants came to the clinic (Glycemic Index Laboratories, Toronto, Canada) in the morning after a 10-14 h overnight fast. After being weighed and having two fasting blood samples obtained by finger-prick 5 min apart, the participant then consumed a study meal within 15 min. At the first bite a timer was started and additional blood samples were taken at 15, 30, 45, 60, 90 and 120 min after the start of the study meal. Before and during the test, a blood glucose test record was filled out with the participants initials, ID number, date, body weight, study meal, time they started eating, time it took to eat, time and composition of last meal, and any unusual activities. Participants remained seated quietly during the 2 h of the test. After completing the test, participants were offered a snack and then allowed to leave.

### Study meals

Each participant underwent a total of 15 separate study meals consisting of 3 white bread control meals and 12 dried fruit test meals (Table 1). The test meals included each of the 4 dried fruits (dates, apricots, raisins, sultanas) alone (GI effect), displacing half the available carbohydrate in white bread (displacement effect), or providing a small ‘catalytic’ dose (7.5 g) of fructose added to white bread (‘catalytic’ fructose effect). Each study meal was designed to contain a total of 50 g of available carbohydrate with the exception of the study meals testing the ‘catalytic’ fructose effect. The’catalytic’ dose of 7.5 g of fructose was derived from both free fructose and fructose bound in sucrose. The nutrient content of the study meals was analyzed by Merieux/Silliker (Toronto, Canada) using the Association of Official Agricultural Chemists approved methods and is provided in Table 2.

**Table 1:** Description of the study meals

Dried fruit test meals			Control meals
GI effect	Displacement effect	‘Catalytic’ fructose effect	
Dates (50 g avCHO)	WB (25 g avCHO) + Dates (25 g avCHO)	WB (50 g avCHO) + Dates (7.5 g fructose)	WB (50 g avCHO)
Apricots (50 g avCHO)	WB (25 g avCHO) + Apricots (25 g avCHO)	WB (50 g avCHO) + Apricots (7.5 g fructose)	WB (50 g avCHO)
Raisins (50 g avCHO)	WB (25 g avCHO) + Raisins (25 g avCHO)	WB (50 g avCHO) + Raisins (7.5 g fructose)	WB (50 g avCHO)
Sultanas (50 g avCHO)	WB (25 g avCHO) + Sultanas (25 g avCHO)	WB (50 g avCHO) + Sultanas (7.5 g fructose)	

*avCHO* available carbohydrate, *GI* glycemic index, *WB* white bread

**Table 2:** Nutrient content of the study meals^a^

Study Meal	Weight (g)	Protein (g)	Fat (g)	Total CHO (g)	Fibre (g)	Available CHO (g)	Fructose (g)^b^	Glucose (g)	Sucrose (g)
*GI effect*									
Dates (50 g avCHO)	77.8	3.1	0.6	57.9	7.9	50.0	13.1	13.6	23.1
Apricots (50 g avCHO)	81.4	3.6	0.3	53.2	3.2	50.0	9.1	13.1	7.3
Raisins (50 g avCHO)	64.8	2.7	0.7	53.1	3.1	50.0	23.0	21.8	0.4
Sultanas (50 g avCHO)	67.0	2.7	1.5	52.7	2.7	50.0	22.7	22.2	<0.1
*Displacement effect*									
WB (25 g avCHO)^c,d^	57.8	4.5	0.9	27.6	1.5	26.1	–	–	0.7
Dates (25 g avCHO)	38.9	1.5	0.3	29.0	4.0	25.0	6.6	6.8	11.6
Apricots (25 g avCHO)	40.7	1.8	0.2	26.6	1.6	25.0	4.6	6.6	3.7
Raisins (25 g avCHO)	32.4	1.4	0.3	26.5	1.5	25.0	11.5	10.9	0.2
Sultanas (25 g avCHO)	33.5	1.3	0.8	26.4	1.4	25.0	11.4	11.1	<0.1
*‘Catalytic’ fructose effect*									
WB (50 g avCHO)^c,d^	115.6	9.0	1.8	55.2	3.0	52.2	–	–	1.4
Dates (7.5 g fructose)	23.6	0.9	0.2	17.6	2.4	15.2	4.0	4.1	7.0
Apricots (7.5 g fructose)	47.8	2.1	0.2	31.2	1.9	29.4	5.4	7.7	4.3
Raisins (7.5 g fructose)	20.9	0.9	0.2	17.2	1.0	16.2	7.4	7.0	0.1
Sultanas (7.5 g fructose)	22.1	0.9	0.5	17.4	0.9	16.5	7.5	7.3	0.0
*Control*									
WB (50 g avCHO)^c,d^	115.6	9.0	1.8	55.2	3.0	52.2	–	–	1.4

*avCHO* available carbohydrate, *CHO* carbohydrate, *GI* glycemic index, *WB* white bread

^a^Maltose and lactose content were also analyzed and were both undetectable across all of the dried fruits

^b^Fructose amount reported in this column is free fructose. Total fructose amount can be derived using the following formula: total fructose = fructose + (sucrose/2)

^c^Although the fructose and glucose content was not measured for white bread, based on the recipe it should not contain any fructose or glucose

^d^White bread was designed to contain 25 g avCHO in study meals assessing the displacement effect and 50 g avCHO in the control and study meals assessing the ‘catalytic’ fructose effect. The analyzed amounts reported in this table slightly differ due to inherent variations within nutrient concentrations of the ingredients used in the preparation of the white bread

### Blood sample collection and analysis

Each finger-prick sample consisted of a total of 2–3 drops of capillary blood collected into flat-bottomed 5 ml plastic tubes with a push cap containing a small amount of sodium fluoride and potassium oxalate as an anticoagulant and preservative. The samples were mixed by rotating the tube vigorously and then refrigerated during the testing session. After completion of the test session, samples were stored at −20 °C prior to blood glucose analysis. Within five days of collection the blood glucose analysis was conducted using a YSI model 2300 STAT analyzer (Yellow Springs, OH) by a lab technician blinded to the treatment allocation.

### Primary outcome

The primary outcome was GI, which was determined using the ISO method (ISO 26642:2010) and adjusted to the glucose scale, where GI of glucose = 100 and white bread = 71. Although the ISO method does not allow for mixed meals (displacement effect, ‘catalytic’ fructose effect) or meals containing > 50 g of available carbohydrate (‘catalytic’ fructose effect), we used this outcome measure for ease of comparison with the GI of dried fruit alone (GI effect).

The GI of the test meals was calculated by expressing each participant’s incremental area under the blood glucose curve (iAUC) values for the test meal as a percentage of the same participant’s mean iAUC for the three white bread control meals. The iAUC values were calculated using the trapezoidal rule, ignoring the area below fasting. For the purpose of the iAUC calculation, fasting glucose was taken to be the mean of the first measurement of the glucose concentration at times -5 and 0 min. GI values > 2*standard deviation (SD) above the mean were excluded and replaced by the mean of the remaining values. GI and iAUC values were expressed as mean ± SEM.

Using the *t*-distribution and assuming an average coefficient of variation (CV) of within individual variation of iAUC values of 25%, *n* = 10 participants had 80% power to detect a 33% difference in iAUC with 2-tailed *P* < 0.05.

### Statistical analysis

Statistical analyses were conducted using SAS software (SAS Inst. Version 8.2; Gary, NC). Data were entered into a spreadsheet by two different individuals and the values compared to assure accurate transcription. Pairwise differences in GI and iAUC between the white bread control and the three mechanisms (GI effect; displacement effect; ‘catalytic’ fructose effect) for each of the 4 dried fruits (dates, apricots, raisins, sultanas) was assessed by the Dunnett’s test, with *P* < 0.05 considered statistically significant.

## Results

**Fig. 1:**
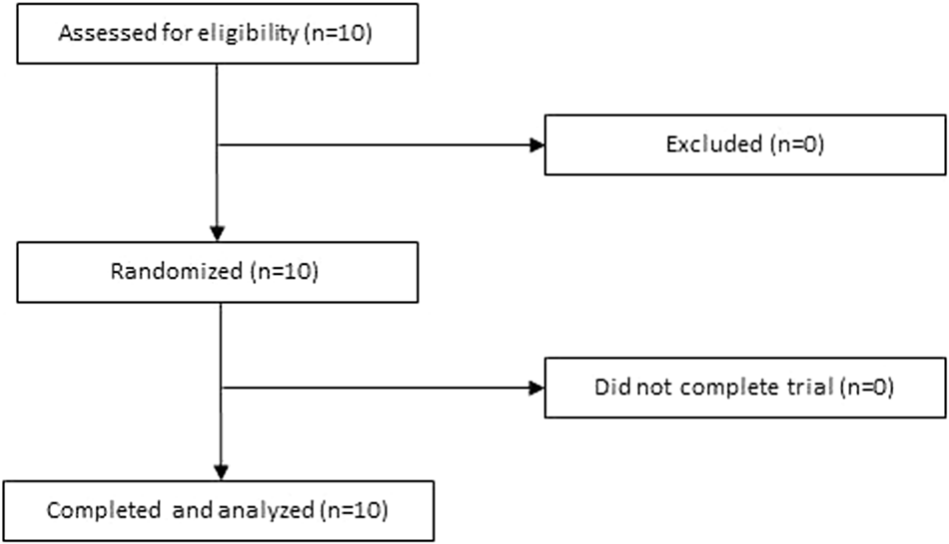
CONSORT flow of participants diagram

A total of 10 participants (7 men; 3 women), with a mean ± SD age and BMI of 39 ± 12 years and 25.3 ± 2.3 kg/m², respectively, were recruited and completed the study between November and December, 2016 (Fig. 1, Table 3).

**Table 3:** Participant characteristics

Characteristic	Mean ± SD or No.
*n*	10
Age (years)	39 ± 12
Sex	
Men	7
Women	3
Race/Ethnicity	
Caucasian	5
Chinese	1
South East Asian	1
Black	1
Korean	1
Mixed	1
Weight (kg)	75.3 ± 12.3
BMI (kg/m²)	25.3 ± 2.3

### Within participant variation of reference food

The mean within CV of the iAUC values after the 3 white bread control meals was 14.6%, which is considered to be satisfactory (values < 30% are considered satisfactory (ISO 26642:2010)).

### Postprandial glycemic responses and glycemic index

Table 4 shows the GI of the 4 dried fruits and their effects on postprandial glycemic responses through the three assessed mechanisms (GI effect; displacement effect; ‘catalytic’ fructose effect). All dried fruits lowered the postprandial glycemic response and had a GI below that of white bread; however, only the dried apricots (GI = 42 ± 5), raisins (GI = 55 ± 5), and sultanas (51 ± 4) showed a significant GI effect (*P* < 0.05). When displacing half the available carbohydrate in white bread, all dried fruit lowered the postprandial glycemic response. However, only dried apricots (GI = 57 ± 5) significantly reduced the glycemic response through the displacement effect (*P* = 0.025). None of the dried fruits showed a beneficial ‘catalytic’ fructose effect.

**Table 4:** Postprandial glycemic responses and glycemic index (*n* = 10)

Study meal	iAUC				Glycemic index^a,b^				
	Mean ± SEM	Test vs. Control estimate	% Change	*P*-value	Mean ± SEM	GI classification^c^	Test vs. Control estimate	% Change	*P*-value
*GI effect*									
Dates (50 g avCHO)	205.9 ± 32.4	−7.8	−3.6	0.983	67.6 ± 6.3	Medium	−3.4	−4.8	0.909
Apricots (50 g avCHO)	117.7 ± 14.3	−96.1	−45.0	<0.0001*	41.5 ± 5.4	Low	−29.5	−41.5	<0.0001*
Raisins (50 g avCHO)	163.4 ± 24.3	−50.3	−23.5	0.045*	54.7 ± 4.7	Low	−16.3	−23.0	0.011*
Sultanas (50 g avCHO)	167.1 ± 25.1	−46.7	−21.8	0.069	50.6 ± 4.0	Low	−20.4	−28.7	0.001*
*Displacement effect*									
WB (25 g avCHO) + Dates (25 g avCHO)	193.8 ± 23.6	−20.0	−9.3	0.588	61.8 ± 3.1	Medium	−9.2	−13.0	0.219
WB (25 g avCHO) + Apricots (25 g avCHO)	165 ± 18.4	−48.7	−22.8	0.023*	56.6 ± 4.8	Medium	−14.4	−20.2	0.025*
WB (25 g avCHO) + Raisins (25 g avCHO)	183.9 ± 17.1	−29.9	−14.0	0.247	64.3 ± 4.5	Medium	−6.7	−9.4	0.491
WB (25 g avCHO) + Sultanas (25 g avCHO)	187.4 ± 19.9	−26.4	−12.4	0.348	65.1 ± 5.0	Medium	−5.9	−8.2	0.601
‘Catalytic’ fructose effect									
WB (50 g avCHO) + Dates (7.5 g fructose)	225.6 ± 29.2	11.9	5.6	0.959	76.4 ± 6.4	High	5.4	7.6	0.892
WB (50 g avCHO) + Apricots (7.5 g fructose)	223.8 ± 23.0	10.0	4.7	0.977	77.3 ± 6.3	High	6.3	8.9	0.830
WB (50 g avCHO) + Raisins (7.5 g fructose)	230.6 ± 36.9	16.8	7.9	0.874	76.7 ± 5.3	High	5.7	8.0	0.872
WB (50 g avCHO) + Sultanas (7.5 g fructose)	279.8 ± 37.0	66.1	30.9	0.022*	94.7 ± 8.8	High	23.7	33.4	0.014*
*Control*									
WB (50 g avCHO)	213.7 ± 25.7	–	–	–	71 ± 0.0	High	–	–	–

*avCHO* available carbohydrate, *GI* glycemic index, *iAUC* incremental area under the blood glucose curve, *WB* white bread

^a^GI values reported in this table consist of those after outliers were excluded and adjusted to the glucose scale

^b^Although the ISO method (ISO 26642:2010) does not allow for mixed meals (displacement effect, ‘catalytic’ fructose effect) or meals containing > 50 g of available carbohydrate (‘catalytic’ fructose effect), we used this outcome measure for ease of comparison with the GI of dried fruit alone (GI effect)

^c^High: GI ≥ 70; Medium: 56 ≥ GI ≤ 69; Low: GI ≤ 55 (ISO 26642:2010)

*Significantly different from control (*P* < 0.05).

## Discussion

The present study demonstrates that dried fruits (dates, apricots, raisins, and sultanas) have a low to medium GI. When displacing half of the available carbohydrate in white bread, all of the dried fruits lowered the GI, with dried apricots showing a significant displacement effect. None of the dried fruits showed a beneficial ‘catalytic’ fructose effect.

### Findings in relation to other studies and potential mechanisms

Although there are a limited number of trials assessing the effect of dried fruits on cardiometabolic health outcomes, our findings are consistent with previous trials showing dried fruit to have a low to medium GI^1–7^ and to lower postprandial glycemia^3,6–11^. Several potential mechanisms may explain these findings. The low to medium GI and source of dietary fibre of dried fruits (~3–8 g of fibre per ¼ cup or 60 mL, which is equivalent to one Canada Food Guide serving^23^) are factors that have both been shown to have benefits for glycemic control and diabetes risk reduction in randomized controlled trials^24–27^ and prospective cohort studies^28–30^. Their viscous fibre and whole food matrix are thought to be other contributors to lowering the glycemic response. Dried fruits are also a good source of phytochemicals (e.g., phenolic acids, flavonoids, carotenoids, etc.)^31^, which may play a role in modifying blood glucose and diabetes risk^32^. Another potential mechanism may relate to the type of sugar present. Fructose, a low GI sugar, comprises 23–50% of the total sugar content of dried fruits^33,34^. In systematic reviews and meta-analyses of controlled feeding trials, we demonstrated that small doses of fructose (defined as ≤ 36 g/day based on the following: 3 meals at ≤ 10 g/meal and 2 snacks at ≤ 3 g/snack)^35^ and larger doses of fructose (median, 60 g/day)^36^ in exchange for other carbohydrate sources decreased HbA_1c_ levels by 0.4 and 0.53%, respectively. This level of reduction exceeds the clinically meaningful threshold of 0.3% proposed by the U.S. FDA for the development of new oral anti-hyperglycemic agents^37^. In terms of our findings regarding a displacement effect of dried fruit, we expect this would also be observed with other high GI carbohydrate foods. The addition of pistachios to a number of commonly consumed high GI carbohydrate foods (bread, pasta, rice, potato) was found to attenuate the glycemic response in healthy individuals (*n* = 10), all of which were statistically significant except for potato^12^. To our knowledge, only one randomized trial has been conducted where 4 different dried fruits (raisins, apples, jujubes, and apricots) were used to displace half the available carbohydrate of white rice in healthy individuals (*n* = 11)^7^. Similar to our findings, all the dried fruits lowered the glycemic response relative to white rice alone, however, none were statistically significant due to high variability^7^. It would appear that a larger number of participants would be required to consistently observe a significant displacement effect with dried fruit.

### Limitations

This study has several limitations. First, the results of this acute feeding trial cannot be translated into long-term benefits. Second, the small sample size of 10 participants, although validated, makes these findings less precise and generalizable. Third, the ISO method for determining GI was not followed for the study meals assessing the displacement and ‘catalytic’ fructose effect mechanisms as they consisted of a mixed meal and/or >50 g of available carbohydrate. The purpose of determining the GI for study meals assessing these two mechanisms was for ease of comparisons with the GI of dried fruit alone (GI effect) and can be regarded as a relative glycemic response. Fourth, an imbalance in available carbohydrate in assessing the ‘catalytic’ fructose effect may have confounded the results. Because the test meals assessing the ‘catalytic’ fructose effect used ‘real world’ whole food sources of fructose from dried fruit rather than fructose alone, there was additional glucose and sucrose contained in these meals. The additional glucose and sucrose would have increased the relative amount of available glycemic carbohydrate (free glucose or glucose from the hydrolysis of sucrose and starch) in the dried fruit test meal compared with that in the white bread control meal (starch alone), resulting in a higher glycemic response, which may have offset any ‘catalytic’ effect of fructose. Future trials should consider standardizing the amount of available glycemic carbohydrate in study meals that assess the effect of ‘catalytic’ doses of fructose from whole foods.

### Implications

Our study aimed to assess the effect of the most commonly consumed dried fruits. According to estimates from the International Nut and Dried Fruit Foundation, dried grapes (including raisins and sultanas), followed by dates, were the most highly consumed dried fruits in both high- and middle-income countries^38^. Estimates were also provided for dried apricots, prunes, and dried figs, which were consumed at lower levels relative to dried grapes and dates^38^. Dried fruits, which can be processed using various drying methods to extend their shelf life as dried fruits,  are nutritionally equivalent to fresh fruits in smaller serving sizes^31^. This property makes dried fruit  easier to store and distribute throughout the year and provides a healthier alternative to high GI food products^31^. Given that a small percentage of the U.S. population consumes dried fruits (7%) (41) and the observed benefits of dried fruits on postprandial glycemic response levels, there is opportunity to use them in combination with high GI foods to lower their GI and help with blood glucose management. Although we predict that the use of other dried fruits to displace available carbohydrate from other high GI foods will produce similar findings to ours, more randomized trials are needed in this area to confirm this.

## Conclusions

The results of this study show that dried fruit have a lower GI than white bread and can lower the glycemic response of white bread through displacement of half of the available carbohydrate. Longer and larger randomized trials are needed to confirm whether dried fruit can contribute to sustainable improvements in glycemic control, as well as assess whether other types of dried fruits and displacement of available carbohydrate from other high GI foods will show similar findings. Overall, these findings will help stimulate important industry innovation and improve the design of future clinical investigations that will potentially lead to the use of dried fruits as an effective tool to modify the glycemic response of high carbohydrate foods.
